# Plastic/Ductile Bulk 2D van der Waals Single‐Crystalline SnSe_2_ for Flexible Thermoelectrics

**DOI:** 10.1002/advs.202203436

**Published:** 2022-08-21

**Authors:** Tingting Deng, Zhiqiang Gao, Pengfei Qiu, Tian‐Ran Wei, Jie Xiao, Genshui Wang, Lidong Chen, Xun Shi

**Affiliations:** ^1^ School of Chemistry and Materials Science Hangzhou Institute for Advanced Study University of Chinese Academy of Sciences Hangzhou 310024 China; ^2^ State Key Laboratory of High Performance Ceramics and Superfine Microstructure Shanghai Institute of Ceramics Chinese Academy of Sciences Shanghai 200050 China; ^3^ Center of Materials Science and Optoelectronics Engineering University of Chinese Academy of Sciences Beijing 100049 China; ^4^ School of Physical Science and Technology ShanghaiTech University Shanghai 201210 China; ^5^ State Key Laboratory of Metal Matrix Composites School of Materials Science and Engineering Shanghai Jiao Tong University Shanghai 200240 China

**Keywords:** flexible thermoelectrics, plastic/ductile semiconductors, single‐crystalline, SnSe_2_

## Abstract

The recently discovered ductile/plastic inorganic semiconductors pave a new avenue toward flexible thermoelectrics. However, the power factors of current ductile/plastic inorganic semiconductors are usually low (below 5 µW cm^−1^ K^−2^) as compared with classic brittle inorganic thermoelectric materials, which greatly limit the electrical output power for flexible thermoelectrics. Here, large plasticity and high power factor in bulk two‐dimensional van der Waals (2D vdW) single‐crystalline SnSe_2_ are reported. SnSe_2_ crystals exhibit large plastic strains at room temperature and they can be morphed into various shapes without cracking, which is well captured by the inherent large deformability factor. As a semiconductor, the electrical transport properties of SnSe_2_ can be readily tuned in a wide range by doping a tiny amount of halogen elements. A high power factor of 10.8 µW cm^−1^ K^−2^ at 375 K along the in‐plane direction is achieved in plastic single‐crystalline Br‐doped SnSe_2_, which is the highest value among the reported flexible inorganic and organic thermoelectric materials. Combining the good plasticity, excellent power factors, as well as low‐cost and nontoxic elements, bulk 2D vdW single‐crystalline SnSe_2_ shows great promise to achieve high power density for flexible thermoelectrics.

## Graphical abstract

Bulk 2D van der Waals (vdW) single‐crystalline SnSe_2_ is reported as a novel inorganic plastic/ductile thermoelectric (TE) material. It exhibits good deformability and excellent power factor at room temperature. Combining the low cost and nontoxic elements, plastic bulk 2D vdW single‐crystalline SnSe_2_ is very promising for fabricating the high power density flexible TE device.

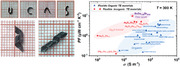

## 1 Introduction

Flexible thermoelectric (TE) technology can convert body heat into electricity to power wearable electronics, which is attracting great interests from both academia and industry.^[^ ^1^, ^2^, ^3^, ^4^, ^5^ ^]^ Flexible TE technology requires high performance flexible TE materials to fabricate high power density flexible TE device. The performance of TE materials can be evaluated by the figure‐of‐merit *zT* = *S* ^2^ *σT*/*κ*, where *S*, *σ*, *κ*, and *T* are Seebeck coefficient, electrical conductivity, thermal conductivity, and absolute temperature, respectively.^[^ ^6^, ^7^, ^8^, ^9^ ^]^ The magnitude of *S* ^2^ *σ*, defined as power factor (*PF*), directly determines the power density of the flexible TE device.^[^ ^10^, ^11^, ^12^, ^13^ ^]^

Recently, the discovery of abnormal plasticity/ductility in Ag_2_S, ZnS crystals (in darkness), and InSe crystals opens a new avenue to fabricate flexible TE materials.^[^ ^14^, ^15^, ^16^ ^]^ Being different with the brittle state‐of‐the‐art TE materials (e.g., Bi_2_Te_3_, PbTe, and SiGe), they exhibit abnormal metal‐like plasticity and deformability at room temperature. For example, Ag_2_S can endure an engineering strain above 20% in the bending test and 4.2% in the uniaxial tensile test.^[^ ^15^ ^]^ Single‐crystalline InSe exhibits ~80% compression strain along and perpendicular to the *c* axis and it can be morphed into different shapes at room temperature.^[^ ^16^ ^]^ Based on these plastic/ductile inorganic semiconductors, a series of high performance flexible inorganic TE materials have been developed, such as Ag_2_S_0.5_Se_0.45_Te_0.05_ with *zT* = 0.44 at 300 K,^[^ ^4^ ^]^ Ag_20_S_7_Te_3_ with *zT* = 0.8 at 600 K,^[^ ^17^ ^]^ Ag_2_Te_0.6_S_0.4_ with *zT* = 0.7 at 573 K,^[^ ^18^ ^]^ Ag_3.95_STe with *zT* = 0.97 at 623 K,^[^ ^19^ ^]^ and (Ag_0.2_Cu_0.785_)_2_S_0.7_Se_0.3_ with *zT* = 0.95 at 800 K.^[^ ^20^ ^]^ Although the *PF* values of Ag_2_S‐based TE materials (usually below 5 µW cm^−1^ K^−2^) are still much lower than those of classic brittle inorganic TE materials,^[^ ^21^, ^22^, ^23^, ^24^, ^25^, ^26^, ^27^ ^]^ the flexible inorganic TE device made of the Ag_2_S‐based materials already exhibits superior normalized maximum power density that are orders of magnitude higher than flexible organic TE devices, showing the great potential to be used in wearable electronics.

**Figure 1:**
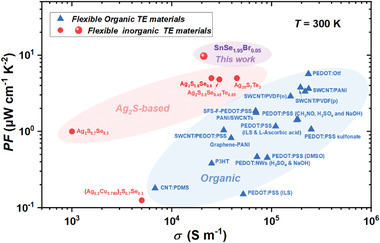
Room‐temperature power factor *PF* as a function of electrical conductivity *σ* for flexible inorganic vdW crystal SnSe_2_‐based TE materials, flexible inorganic Ag_2_S‐based TE materials,^[^ ^4^, ^17^, ^18^, ^19^, ^20^, ^28^ ^]^ and typical flexible organic TE materials.^[^ ^1^, ^12^, ^29^, ^30^, ^31^ ^]^

The achievements in Ag_2_S‐based TE materials and devices inspire the great enthusiasm in finding new plastic inorganic semiconductors toward flexible thermoelectrics, particularly for those with high *PF* near room temperature. In this work, we report that bulk van der Waals (vdW) single‐crystalline SnSe_2_ also exhibits good plasticity at room temperature, which can be well understood by its inherent large deformability factor. Upon doping a tiny amount of halogen elements, the plastic single‐crystalline SnSe_2_ demonstrates a high *PF* of 10.8 µW cm^−1^ K^−2^ and a *zT* of 0.09 at 375 K along the in‐plane direction. The room‐temperature *PF* of single‐crystalline SnSe_2_ is about two times of the maximum *PF* values of the flexible organic TE materials and flexible inorganic Ag_2_S‐based TE materials reported before (**Figure** **1**). This study promises a new plastic inorganic semiconductor toward flexible thermoelectrics.

## 2 Results and Discussion

**Figure 2:**
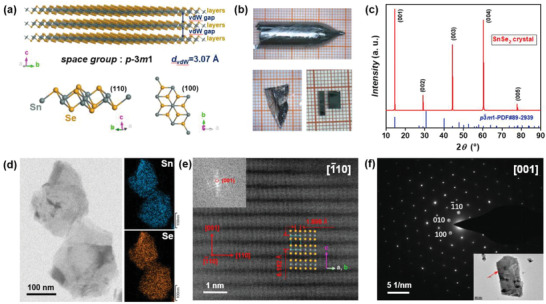
a) Crystal structure of bulk 2D vdW SnSe_2_ and the projections on the (110) and (001) planes. b) Optical images of as‐prepared SnSe_2_ ingot and the single crystals peeled off the ingot. c) XRD pattern performed on the cleaved surface along the in‐plane direction. d) STEM images with elemental mapping of a SnSe_2_ sheet. e) HAADF‐STEM image along [$\bar{1}$10] zone axis. f) SAED pattern along axis [001].

SnSe_2_ is a typical 2D vdW crystal with the space group of *p‐3m1* (**Figure** **2**a). The lattice parameters are *a* = *b* = 3.811 Å, *c* = 6.137 Å; *α* = *β* = 90°, *γ* = 120°. The hexagonal close‐packed [Se‐Sn‐Se] layers are stacked along the *c* axis, forming bulk crystal with the vdW gap about 3.07 Å.^[^ ^32^, ^33^ ^]^ Figure 2b shows the as‐grown bulk SnSe_2_ crystal. Due to the weak chemical bonding between the adjacent layers, the bulk SnSe_2_ crystal is easy to cleave, forming smooth surfaces (Figure 2b).^[^ ^34^, ^35^ ^]^ As shown in Figure 2c, only the (00*l*) (*l* = 1, 2, 3, …) diffraction peaks can be detected on the cleaved surface, confirming the high quality of the crystals. Energy dispersive spectroscopy (EDS) mapping indicates that Sn and Se elements are homogeneously distributed inside the matrix (Figure 2d). The aberration‐corrected high‐angle annular dark‐field scanning transmission electron microscopy (HAADF‐STEM) image along [$\bar{1}$10] zone axis shows that the atomic arrangements match well with the hexagonal layered structure of SnSe_2_ (Figure 2e). Selected area electron diffraction (SAED) patterns shown in Figure 2f and Figure S1 in the Supporting Information well match the hexagonal structure with [001] and [110] zone axes, respectively. Furthermore, the {100} plane spacing (*d*) is calculated with a value of 3.338 Å, which is comparable with the value obtained from the lattice parameter (3.300 Å) based on *d =* 1*/* $\sqrt{\frac{4}{3}\;\frac{\left(h^{2}+hk+k^{2}\right)}{a^{2}}+{\left(\frac{l}{c}\right)}^{2}}$.

**Figure 3:**
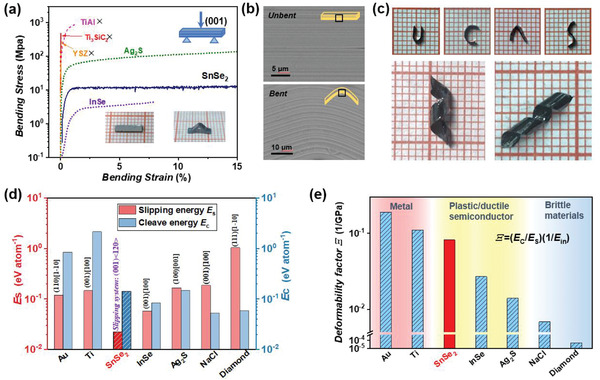
a) Engineering stress–strain curve of three‐point bending test performed on bulk 2D vdW SnSe_2_ single crystal. The data of bulk 2D vdW InSe single crystal, Ag_2_S, TiAl, Ti_3_SiC_2_, and YSZ are also included for comparison. The inserts show the optical images of the SnSe_2_ sample before and after testing. b) Cross‐section SEM images of unbent and bent plastic single‐crystalline SnSe_2_. c) SnSe_2_ single crystals morphed into various shapes without cracking. Calculated d) slipping energy barrier *E*_s_, cleavage energy *E*_c_, and e) deformability factors *Ξ* along the slipping system (001)<120> for SnSe_2_. The data of Au, Ti, InSe, Ag_2_S, NaCl, and diamond are also included for comparison.^[^ ^16^ ^]^

Polycrystalline SnSe_2_ is brittle, which cannot withstand any deformation under external stress (Figure S2, Supporting Information). Interestingly and surprisingly, single‐crystalline SnSe_2_ is plastic/ductile at room temperature. **Figure** **3**a shows that SnSe_2_ can endure a large bending strain above 15% without cracking in the three‐point bending test. These large engineering strains are comparable with those of polycrystalline Ag_2_S, single‐crystalline InSe, and most metals, while much higher than those brittle materials (e.g., Ti_3_SiC_2_ and yttria‐stabilized zirconia (YSZ), less than 1%).^[^ ^15^, ^16^ ^]^ The cross‐section scanning electron microscopy (SEM) image confirms that the bent single crystal SnSe_2_ still maintains good integrity in microscale alike that before bending (Figure 3b). Due to the excellent plasticity, the sheets peeled off from a SnSe_2_ single crystal show good deformability. They can be morphed into various shapes without cracking (Figure 3c). It should be noted that such deformability related to plasticity is different with the common flexibility observed in monolayer or few‐layers 2D vdW materials. The latter one describes the elastic, reversible deformability, mostly bendability that highly depends on the thickness. In the multilayers form or bulk form, these materials are easily cracked under stress. Thus, the intrinsic plasticity endows bulk 2D vdW single‐crystalline SnSe_2_ more freedom of machining and integrating in flexible electronics.

The large plasticity and deformability of bulk 2D vdW single‐crystalline SnSe_2_ can be understood based on the deformability factor, which is defined as *Ξ* = (*E*_c_/*E*_s_)(1/*E*_in_), where *E*_s_ is the slipping energy barrier, *E*_c_ is the cleavage energy, and *E*_in_ is the Young's modulus along the slip direction.^[^ ^16^ ^]^ The material with a larger *Ξ* is prone to have better plasticity since the large *E*_c_/*E*_s_ facilitates slipping without fracture while the low *E*_in_ favors elastic bending. Density functional theory (DFT) calculations are performed to obtain *E*_c_, *E*_s_, and *E*_in_ of bulk 2D vdW SnSe_2_. As shown in Figure 3d, the calculated *E*_s_ along the slipping system (i.e., the easiest slipping pathway) (001)<120> for SnSe_2_ is 0.022 eV per atom, much lower than those of most metals and brittle materials (e.g., 0.147 eV per atom for Ti along (001)[100] and 1.044 eV per atom for diamond along (111)[1$\bar{1}$0]). Actually, this value is even lower than that of plastic 2D vdW InSe along (001)[100] and bulk Ag_2_S along (100)[001]. The cleavage energy of SnSe_2_ along the cleave plane (001) is 0.143 eV per atom, which is comparable with brittle materials and plastic semiconductors (e.g., 0.059 eV per atom for diamond along (111), 0.084 eV per atom for InSe along (001) and 0.148 eV per atom for Ag_2_S along (100)). Combining the calculated *E*_in_ (78.77 GPa), the *Ξ* = 0.082 1/GPa is obtained of 2D vdW SnSe_2_. As shown in Figure 3e, this value is much higher than those of brittle NaCl, and diamond, while comparable with those of plastic Ag_2_S and 2D vdW InSe. These results can well explain the good plasticity of 2D vdW single‐crystalline SnSe_2_ observed in experiment. In the polycrystal SnSe_2_, the continuous interlayer gliding requires the coordination from the adjacent grains, which is very difficult due to the random grain orientation and limited slipping system. Thus, the polycrystalline SnSe_2_ has much poorer plasticity than the single crystalline SnSe_2_.

**Figure 4:**
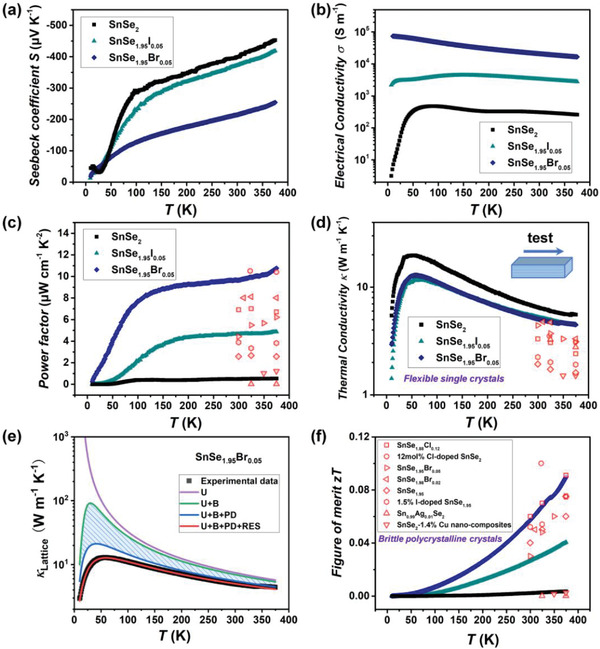
Temperature‐dependent a) Seebeck coefficient *S*, b) electrical conductivity *σ*, c) power factor *PF*, and d) thermal conductivity *κ* of plastic single‐crystalline SnSe_2_ doped by halogen elements. e) Contributions from various phonon scattering mechanisms to the *κ*_Lattice_ of SnSe_1.95_Br_0.05_. U, B, PD, and RES denote the phonon–phonon Umklapp process scattering, grain boundary scattering, point defect scattering, and phonon resonant scattering, respectively. f) Temperature‐dependent TE figure‐of‐merit *zT*. The data for single element doped brittle polycrystalline SnSe_2_‐based samples^[^ ^33^, ^36^, ^37^, ^38^, ^39^, ^40^, ^41^ ^]^ reported before are included in (c), (d), and (f) for comparison.

The TE properties of brittle polycrystalline SnSe_2_ above room temperature have been already extensively investigated by many groups.^[^ ^33^, ^36^, ^37^, ^38^, ^39^, ^40^, ^41^, ^42^, ^43^, ^44^, ^45^, ^46^ ^]^ For flexible thermoelectric, the TE properties near the room temperature are extremely important, but they are rarely studied in single‐crystalline SnSe_2_. Herein, the in‐plane TE properties of plastic bulk 2D vdW single‐crystalline SnSe_2_ below 375 K are systematically studied. As shown in **Figure** **4**a, SnSe_2_ exhibits negative Seebeck coefficients *S*, indicating that the majority carriers are electrons. The *S* increases with increasing temperature, reaching −453 µV K^−1^ at 375 K. The electrical conductivity *σ* of SnSe_2_ first increases with increasing temperature (Figure 4b), reaching a peak value around 50 K, and then decreases at higher temperature. Based on the measured *S* and *σ*, the *PF* is calculated with a value of 0.53 µW cm^−1^ K^−2^ at 375 K (Figure 4c). Likewise, the thermal conductivity *κ* increases with increasing temperature (Figure 4d), reaching a crystalline peak at 50 K, and then decreases at higher temperature due to the strengthened Umklapp process phonon scattering. The *zT* of SnSe_2_ is about 0.004 at 375 K (Figure 4f).

The low Hall carrier concentration (*n*_H_) is the main reason for the low *PF* and *zT* of pristine SnSe_2_ crystal. At 300 K, the *n*_H_ for single‐crystalline SnSe_2_ is 1.6 × 10^18^ cm^−3^, which is much lower than the optimal range (10^19^–10^20^ cm^−3^) for the classic brittle inorganic TE materials. Here we further doped SnSe_2_ with a tiny amount of halogen elements (Br and I) at the Se‐sites. The room‐temperature *n*_H_ is significantly enhanced to 5.6 × 10^18^ cm^−3^ for SnSe_1.95_I_0.05_ and 3.4 × 10^19^ cm^−3^ for SnSe_1.95_Br_0.05_. The Hall carrier mobility (*µ*_H_) values are 40.3 and 41.1 cm^2^ V^−1^ s^−1^ for single‐crystalline SnSe_1.95_I_0.05_ and SnSe_1.95_Br_0.05_, respectively, which are comparable with those of typical TE materials.^[^ ^47^ ^]^ Consequently, the *σ* and *PF* are significantly increased over the entire measured temperature range. A maximum *PF* of 10.8 µW cm^−1^ K^−2^ is achieved for single‐crystalline SnSe_1.95_Br_0.05_ at 375 K, which is higher than the *PF* values of single element doped brittle polycrystalline SnSe_2_ samples reported before. As shown in Figure 1, the room‐temperature *PF* value of single‐crystalline SnSe_1.95_Br_0.05_ is about two times of the maximum *PF* values of the flexible organic TE materials and flexible inorganic Ag_2_S‐based TE materials reported before. Such high *PF* will facilitate the fabrication of high power density TE device.

Doping halogen elements at the Se‐sites also reduces the *κ*, which is originated from the lowered lattice thermal conductivity (*κ*_Lattice_) by additional point defect (PD) scattering introduced by halogen dopant (Figure S3, Supporting Information). To illustrate this, we fit the *κ*_Lattice_ of SnSe_2_ and SnSe_1.95_Br_0.05_ below 375 K based on the Debye–Callaway model^[^ ^48^, ^49^, ^50^ ^]^

(1) $$\kappa_{\mathrm{Lattice}}=\frac{k_{B}}{2\pi^{2}\nu_{\mathrm{avg}}}\;{\left(\frac{k_{B}T}{\hbar }\right)}^{3}\int_{0}^{\Theta_{D}/T}\tau_{C}\frac{x^{4}e^{x}}{{\left(e^{x}-1\right)}^{2}}dx$$

where *x* = ℏ*ω*/*k*_B_ *T* is the reduced phonon energy, *ω* is the phonon frequency, ℏ is the reduced Plank constant, *k*_B_ is the Boltzmann constant, *ν*_avg_ is the average acoustic velocity (2170 m s^−1^ for SnSe_2_), and *Θ*_D_ is the Debye temperature (218.7 K for SnSe_2_).^[^ ^33^ ^]^ *τ*_C_ is the relaxation time, which can be expressed as

(2) $$\tau_{C}^{-1}=\frac{\nu_{\mathrm{avg}}}{L_{0}}\;+A\omega^{4}+B\omega^{2}Te^{-\Theta_{D}/3T}+\frac{C\omega^{2}}{{\left(\omega_{0}^{2}-\omega^{2}\right)}^{2}}$$

The terms on the right of Equation (2) represent grain‐boundary scattering (GB), point defect scattering (PD), Umklapp process (U), and phonon resonant scattering (RES), respectively.

*L*_0_ is the grain size, *ω*_0_ is the resonant frequency, and *A*, *B* and *C* are the fitting parameters for point defect scattering, phonon–phonon Umklapp scattering, and phonon resonant scattering, respectively. As shown in Figure 4e and Figure S4 in the Supporting Information, the final fitted *κ*_Lattice_ curves (red solid curve) are well consistent with the experimental data (black square symbols). Based on the fitting parameters listed in Table S1 in the Supporting Information, the contribution of each term is plotted in Figure 4e and Figure S4 in the Supporting Information. It can be seen that the *κ*_Lattice_ reduction caused by the point defect scattering in SnSe_1.95_Br_0.05_ (Figure 4e) is larger than that in SnSe_2_ (Figure S4, Supporting Information), particularly above 200 K. The fitting parameter *A* (0.127 × 10^−41^ s^3^) for SnSe_1.95_Br_0.05_ is higher than that (0.047 × 10^−41^ s^3^) for SnSe_2_, which further corroborates the strengthened point defect scattering upon doping halogen dopant in SnSe_2_. However, the *κ* for SnSe_1.95_Br_0.05_ crystal, 5.1 W m^−1^ K^−1^ at 300 K, is still higher than those of brittle polycrystalline SnSe_2_‐based samples reported before (Figure 4d). Finally, upon doping Br, the *zT* is enhanced to 0.09 at 375 K. As shown in Figure 4f, this *zT* value is comparable with the best values reported for single element doped polycrystalline SnSe_2_ samples at the similar temperature range.

**Figure 5:**
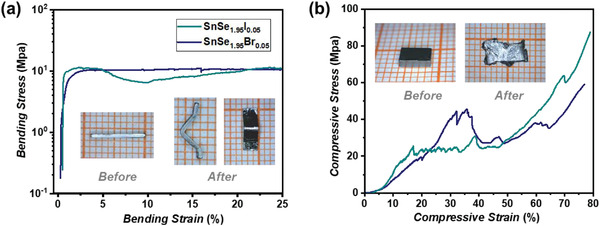
Engineering stress–strain curves of a) three‐point bending test and b) compression test performed on bulk plastic single‐crystalline SnSe_1.95_I_0.05_ and SnSe_1.95_Br_0.05_. The insets show the optical images of SnSe_1.95_Br_0.05_ before and after testing. The serrations in (b) are caused by the dislocation nucleation, crosslink, and break off.

Beyond the significantly improved *PF* and *zT*, the halogen elements doped single‐crystalline SnSe_2_ samples still maintain good plasticity alike the pristine SnSe_2_ at room temperature. As shown in **Figure** **5**a, the single‐crystalline SnSe_1.95_I_0.05_ and SnSe_1.95_Br_0.05_ can endure a large bending strain above 25% along the in‐plane direction without cracking in the three‐point bending test. In the compression test, they exhibit >70% engineering strain when compressed along the *c*‐axis (Figure 5b). After the compression test, these doped samples are spread into a lamina but still maintains the integrity. This feature is very important for the stable service of flexible thermoelectrics since the fracture of TE materials is one of the main failure mechanisms for flexible TE devices during the repeatable bending and stretching process.

## 3 Conclusion

In summary, we report that bulk 2D vdW single‐crystalline SnSe_2_ is a promising inorganic plastic/ductile TE material. It exhibits good plasticity and deformability at room temperature, which can be understood by the large deformability factor. Combining the high *PF* and the unique combination of low‐cost and nontoxic elements, plastic bulk 2D vdW single‐crystalline SnSe_2_ is very appealing for fabricating the high power density flexible TE device. This study also sheds light on exploring new inorganic plastic TE materials from the numerous bulk 2D vdW semiconductors.

## 4 Experimental Section

### Crystal Growth

Bulk 2D vdW SnSe_2_ single crystals were grown by the temperature gradient method. High‐purity raw materials of Sn shots (99.999%, Alfa Aesar), Se shots (99.999%, Alfa Aesar), SnI_4_ powders (99.998%, Aladdin), and SnBr_2_ powders (99.2%, Alfa Aesar) were weighed out based on the chemical stoichiometry as designed and sealed in quartz tubes under vacuum (<10^−2^ Pa). Then, the quartz tubes were heated to 1003 K with a rate of 50 K h^−1^ and held at this temperature for 5 h. Finally, the temperature was gradually decreased with a rate of 1–2 K h^−1^ to 823 K and then rapidly decreased to room temperature at a rate of 100 K h^−1^. The obtained single crystal samples were peeled and cut into specific shapes for measurements.

### Characterization

The phase purity and crystal structure of the SnSe_2_ single crystal were determined by X‐ray diffractometer with Cu *K*_*α*_ sources (XRD, D/max‐2550 V, Rigaku, Japan) and transmission electron microscopy (TEM, JEM‐1400, Japan) with the selected area electron diffraction (SAED). The sample morphology was characterized by field emission scanning electron microscopy (FESEM, ZEISS Supra 55, Germany). The elemental distribution was characterized by scanning transmission electron microscopy (STEM, HF5000, HitachiTech, Japan) with energy dispersive X‐ray spectroscopy (EDS, Oxford, UK). The atomic arrangements were characterized by the aberration‐corrected STEM using a high‐angle annular dark‐field derector. Three‐point bending test was performed by using dynamic thermomechanical analyzer (DMA 850, TA Instruments, USA) with a constant loading rate of 0.1 mm min^−1^. Compression tests were performed by using universal testing machine (AGS‐X, Shimadzu, Japan) with a constant loading rate of 0.05 mm min^−1^. For single‐crystalline SnSe_2_, the sample dimensions used for bending test and compression test are about 2 × 0.7 × 8 and 3 × 4 × 1.2 mm^3^, respectively. The electrical conductivity (*σ*), Seebeck coefficient (*S*), and the thermal conductivity (*κ*) were measured from 10 to 375 K by Physical Property Measurement System (PPMS, Quantum Design, USA). The sample dimension used for electrical resistance measurement is about 2 × 1 × 8 mm^3^. The sample dimension used for Seebeck coefficient and thermal conductivity measurement is about 3 × 4 × 1.5 mm^3^.

### Calculation

The density functional theory (DFT) calculations were performed by the projector augmented wave (PAW) method through Vienna ab initio simulation package (VASP) with the Perdew‐Burke‐Ernzerhof (PBE) functional with DFT‐D3 (Becke and Johnson,BJ) vdW correction.^[^ ^51^, ^52^, ^53^, ^54^ ^]^ A plane‐wave cutoff energy of 600 eV and a *k*‐point density of 12 × 12 × 8 were obtained. The cleavage energy *E*_c_ was calculated by the classical slab model. Vacuum layers with different thicknesses were inserted into the supercell with 4 [Se‐Sn‐Se] layers to obtain the energy change per unit atom. The cleavage energy was determined as the energy reaching the saturation. For the calculation of slapping energies, a supercell of 8 [Se‐Sn‐Se] layers and a vacuum layer thickness of 10 Å were adopted. To find the easiest slipping pathway, a mesh of 11 × 11 steps was made in the (001) plane. The slipping energy variation versus the relative positions was calculated for each step. The <120> direction has the lowest energy barrier, thus it was identified as the easiest slipping pathway and (001)<120> was determined as the possible slipping system. The slipping energies were calculated along the (001)<120> slipping system and the maximum energy was defined the slipping energy barrier *E*_s_. The convergence criteria for force relaxation along the direction perpendicular to the slip planes were 0.03 eV Å^−1^. The *E*_in_ for SnSe_2_ was calculated by the online tool of Elastic tensor analysis (ELATE) software (http://progs.coudert.name/elate) with the elastic stiffness tensor *C*_ij_ from Materials Project database (mp‐665).

## Conflict of Interest

The authors declare no conflict of interest.

## Supporting information

Supporting Information

## Data Availability

The data that support the findings of this study are available in the supplementary material of this article.
