# Supplementary material for: Plastic/Ductile Bulk 2D van der Waals Single‐Crystalline SnSe2 for Flexible Thermoelectrics
Source: Adv Sci (Weinh). 2022 Aug 21;9(29):2203436. doi: 10.1002/advs.202203436 (PMC9561768; doi:10.1002/advs.202203436)
Supplement: Supplementary file 1 — Supporting Information [file ADVS-9-2203436-s001.pdf]

ADVANCED SCIENCE
Open Access

Supporting Information

for *Adv. Sci.*, DOI 10.1002/advs.202203436

Plastic/Ductile Bulk 2D van der Waals Single-Crystalline $SnSe_2$ for Flexible Thermoelectrics

Tingting Deng, Zhiqiang Gao, Pengfei Qiu, Tian-Ran Wei, Jie Xiao, Genshui Wang, Lidong Chen and Xun Shi**

Supporting Information

Plastic/ductile bulk 2D van der Waals single-crystalline $SnSe_2$ for flexible thermoelectrics

Tingting Deng#, Zhiqiang Gao#, Pengfei Qiu, Tian-Ran Wei, Jie Xiao, Genshui Wang, Lidong Chen, and Xun Shi

Dr. T. Deng, Dr. P. Qiu, Prof. G. Wang

School of Chemistry and Materials Science, Hangzhou Institute for Advanced Study, University of Chinese Academy of Sciences, Hangzhou 310024, China

Dr. T. Deng, Z. Gao, Dr. P. Qiu, J. Xiao, Prof. L. Chen, Prof. X. Shi

State Key Laboratory of High Performance Ceramics and Superfine Microstructure, Shanghai Institute of Ceramics, Chinese Academy of Sciences, Shanghai 200050, China

Z. Gao, Prof. L. Chen, Prof. X. Shi

Center of Materials Science and Optoelectronics Engineering, University of Chinese Academy of Sciences, Beijing 100049, China

Z. Gao

School of Physical Science and Technology, Shanghai Tech University, Shanghai 201210, China

Dr. T.-R. Wei

State Key Laboratory of Metal Matrix Composites, School of Materials Science and Engineering, Shanghai Jiao Tong University, Shanghai 200240, China

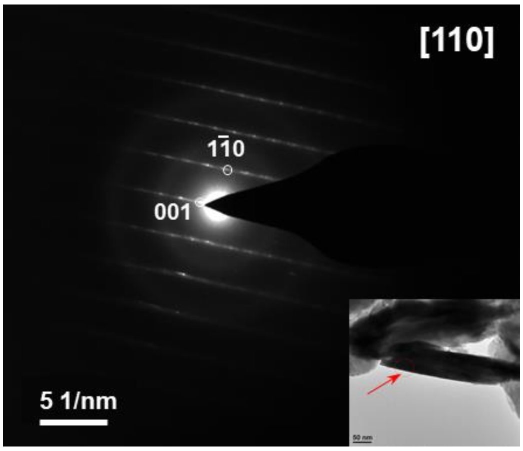

Figure S1. SAED pattern along axis [110] performed on a $SnSe_2$ particle.

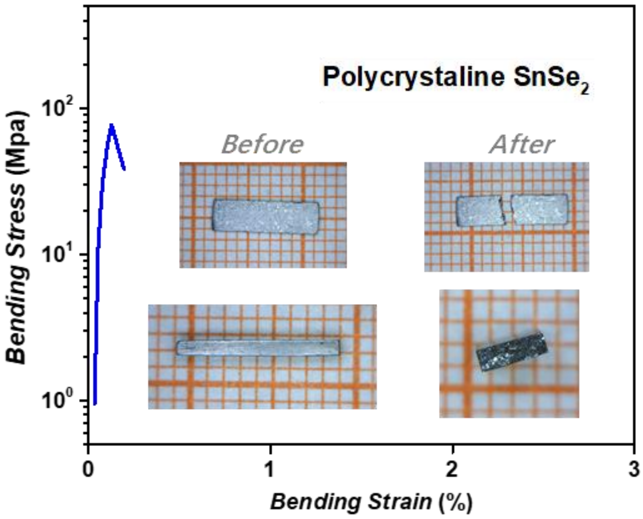

Figure S2. Engineering stress-strain curves of three-point bending test performed on bulk polycrystalline $SnSe_2$. The insets show the optical images of bulk polycrystalline $SnSe_2$ before and after testing. The sample dimension is about $3\times1\times10$ mm^3.

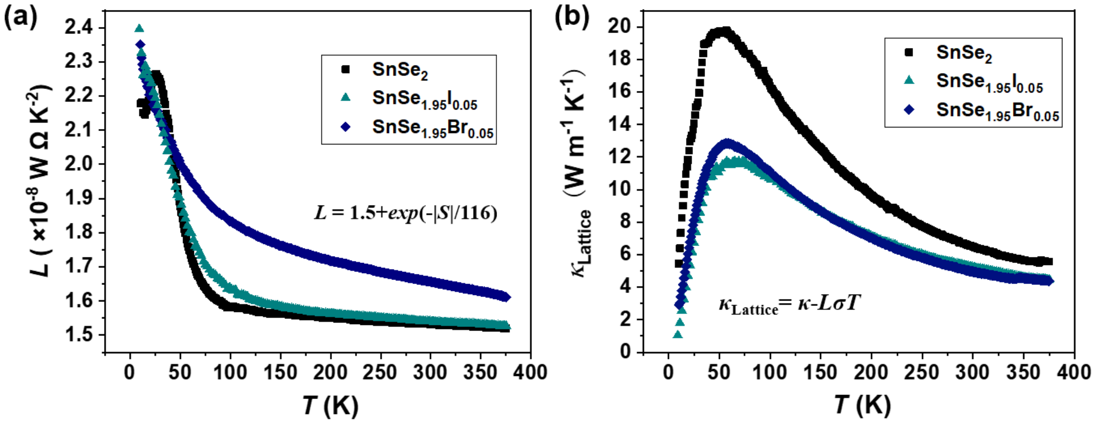

Figure S3. Temperature-dependent (a) Lorenz number L and (b) lattice thermal conductivity $\kappa_{Lattice}$ of plastic single-crystalline $SnSe_2$ doped by halogen elements. The L is estimated by the equation L=1.5+exp (-│S│/116), where S is Seebeck coefficient in μV K^{-1}.[1]

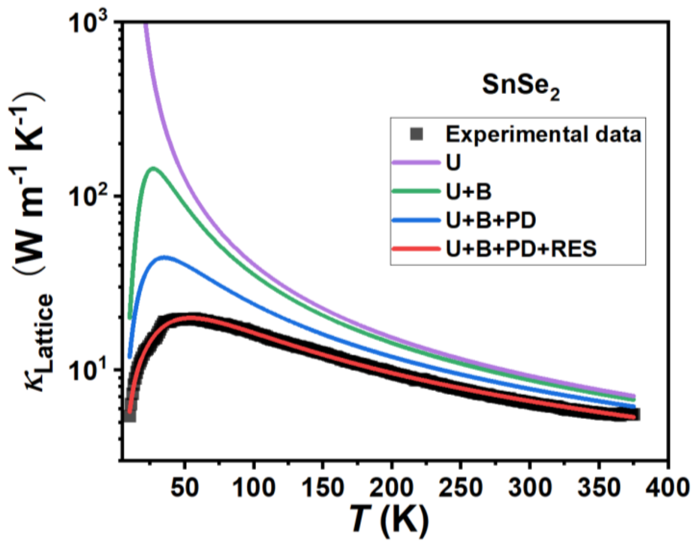

Figure S4. Contributions from various phonon scattering mechanisms to the $\kappa_{Lattice}$ of $SnSe_2$. U, B, PD, and RES denote the phonon–phonon Umklapp process scattering, grain boundary scattering, point defect scattering, and phonon resonant scattering, respectively.

Table S1 Parameters used to fit the lattice thermal conductivity $\kappa_{Lattice}$.

**Fitting parameters**	**$SnSe_2$**	**$SnSe_{1.95}Br_{0.05}$**
v_{avg}/L_0 (10^8 s^{-1})	9.39	18.79
A (10^{-41} s^3)	0.047	0.127
B (10^{-18} s K^{-1})	4.18	5.182
C (10^{36} s^{-3})	0.973	0.1844
ω_0 (THz)	11.99	7.646
R^2	0.99919	0.99964
χ^2	0.18762	0.07421

References

[1] H.-S. Kim, Z. M. Gibbs, Y. Tang, H. Wang, G. J. Snyder, *APL Mater.* **2015**, *3*, 041506.
